# 

**Published:** 1816-07

**Authors:** 


### THE LONDON MEDICAL AND PHYSICAL JOURNAL;

CONTAINING

ORIGINAL CORRESPONDENCE of EMINENT PRACTITIONERS,

AND THE

EARLIEST INFORMATION ON SUBJECTS CONNECTED WITH MEDICINE, SURGERY, CHEMISTRY, PHARMACY, BOTANY, AND NATURAL HISTORY.

*Successively conducted by*

T. BRADLEY, M.D.

A. F. M. WILLICH, M.D.
R. BATTY, M.D.
A. A. NOEHDEN, M.D.
J. ARNEMAN, M.D.
J. P. PFAFF, M.D.
J. ADAMS, M.D.
W. SHEARMAN, M.D.
W. ROYSTON, Esq.

AND

SAMUEL FOTHERGILL, M.D.

Assisted by eminent Gentlemen, in the various Branches of the Profession.

VOL. XXXVI.

FROM JULY TO DECEMBER, 1816.

5142
1816

Et quoniam variant morbi, variabimus artes;
Mille mali species, mille salutis erunt.

*LONDON:*

PRINTED FOR THE PROPRIETORS, BY J. ADLARD, 23, BARTHOLOMEW-CLOSE, AND 39, DUKE-STREET, SMITHFIELD.

PUBLISHED BY J. SOUTER, NO. 1, PATERNOSTER-ROW; AND MAY BE HAD OF ALL BOOKSELLERS.

W1
LO31

[Entered at Stationers' Hall.]

### MONTHLY CATALOGUE OF MEDICAL BOOKS.

ESSAYS on Insanity, Hypochondriasis, and other Nervous Affections. By John Reid, M.D. of the Royal College of Physicians, London. 8vo.

AN Analysis of the Mineral Water of Tunbridge Wells, with some Account of its Medicinal Properties. By Charles Scudamore, M.D. 8vo.

An Essay on the Utility of Blood-letting in Fever. Illustrated by numerous Cases. Second Edition. With a Supplement, containing Cases and Dissection, which occurred in Private Practice. By Thos. Mills, M.D. Licentiate of the King and Queen's College of Physicians, one of the Physicians of St. George's Hospital and Dispensary, and late of the House of Recovery and Fever Hospital, Cork-street, Dublin. 8vo.

Observations on the Harveian Doctrine of the Circulation of the Blood. By George Kerr. 12mo.

*In collecting the List of Publications, some difficulties have occurred, with which we shall not at present trouble our readers; but shall be thankful for information from the authors themselves, or from any other source.*

---

### *NOTICES TO CORRESPONDENTS.*

*A Letter from Bristol informs us, that* M. C., *whose case is mentioned in our last,* " *considering her former ill state of health, is doing as well as may reasonably be expected.*

*We trust* Mr. FULMER *will not be offended at our omitting his grateful reflections on a subject, which, if he peruses our present Number, he will be convinced should be left to the parties themselves. Probably one of them will wish to reserve Mr. Fulmer's opinions for another place.*

*We are concerned* Dr. DICKSON *should feel hurt at any of our remarks on his truly valuable paper. We regret that his letter did not arrive in time for this Number; it shall appear in our next.*

Mr. KERR's *ingenious enquiries and remarks shall appear in our next.*

Mr. LITTLETON's *paper shall be inserted as soon as we can procure an accurate engraving of his coloured drawings. As, in our opinion, his description would be perfectly intelligible without any engravings—certainly without colouring; we submit to him whether one or both might not be omitted. We shall be thankful for his answer; and, by the present specimen, promise ourselves much further information from the remainder of his paper.*

Mr. PARKINSON's *favor shall be attended to; and also* Mr. ATKINSON's.

*We are to acknowledge the receipt of* Mr. WALKER's *Table and Explanation;* Dr. COLLINGWOOD's *cases; and several other papers.*

*We have received* Dr. ABERS's *Prize Dissertation on Croup. In our remarks on which, we shall be as minute and impartial, we trust, as we have ever shown ourselves in the publications of our own country.—We have also to acknowledge the receipt of* Dr. MILLS's *Essay on Blood-letting in Fever; and* Mr. DOUGHTY's *Memoirs of Yellow Fever.*

